# Luminance Contrast Perception in Killer Whales (*Orcinus orca*)

**DOI:** 10.3390/ani15060793

**Published:** 2025-03-11

**Authors:** Ayumu Santa, Koji Kanda, Yohei Fukumoto, Yuki Oshima, Tomoya Kako, Momoko Miyajima, Ikuma Adachi

**Affiliations:** 1 Center for the Evolutionary Origins of Human Behavior, Kyoto University, Inuyama 484-8506, Japan; 2 Port of Nagoya Public Aquarium, Nagoya Port Foundation, Nagoya 455-0033, Japan

**Keywords:** marine mammal, cetacean, killer whale, animal cognition, visual perception, object recognition, contrast, luminance contrast illusion

## Abstract

Cetaceans are highly adapted to the underwater environment, which is very different from the terrestrial environment. For cetaceans with neither high visual acuity nor color vision, contrast may be an important cue for visual object recognition, even in the underwater environment. Contrast is defined as the difference in luminance between an object and its background and is known to be perceived as enhanced by the luminance contrast illusion in humans. The aim of this study was to experimentally investigate whether the enhancement of contrast by the luminance contrast illusion could be observed in killer whales. Luminance discrimination tasks were performed on two captive killer whales, which were required to compare the luminance of two targets presented in monitors through an underwater window and to choose the brighter one. After baseline training, in which the target areas were surrounded by black or white inducer areas, the test condition of gray inducer areas was added. Although there were some individual differences, both individuals showed higher correct response rates for gray inducer conditions than for black and white. The results suggest that contrast was perceived as enhanced by the illusion also in killer whales and may help them to extract the contours of objects.

## Simple Summary

The underwater environment is very different from the terrestrial environment, in which most mammals live. The cognitive abilities of cetaceans (dolphins, porpoises, and whales) have been greatly affected by the characteristics of the underwater environment. While their auditory abilities have been studied extensively, there has not been enough research focused on their visual abilities. This study focused on their visual ability to perceive contrast and aimed to experimentally investigate whether the enhancement of contrast can be observed in killer whales. Luminance discrimination tasks were performed on two captive killer whales, which were required to compare the luminance of two targets presented in monitors through an underwater window and to choose the brighter one. Although there were some individual differences, both individuals showed higher correct response rates when the two targets were surrounded by gray backgrounds than when they were surrounded by black or white backgrounds. The results suggest that contrast was perceived as enhanced in killer whales, as in humans. For killer whales with neither high visual acuity nor color vision, contrast may be an important cue for visual object recognition and may help them to extract the contours of objects even in the underwater environment.

## 1. Introduction

Contrast is defined as the difference in luminance between an object and its background. In humans, contrast plays an important role in visual object recognition [1], along with other visual cues, such as size, color, shape, texture, motion, and so on. Previous studies have shown that the increasing contrast in visual search tasks decreases the average reaction time and the number of fixations per search [2,3,4,5]. A greater difference in luminance between the object and its background makes it easier to find the contours of the object from the background. In addition, it has been reported that humans have the visual ability to perceive contrast more emphatically than it is, which is known as one aspect of the ‘luminance contrast illusion’ [6]. When two areas of different luminance are adjacent, the contrast, which is the difference in luminance between these two areas, is perceived as enhanced due to this illusion. For example, a gray patch surrounded by a dark background is perceived as lighter than it is, and, conversely, the gray patch is perceived as darker when surrounded by a light background. In visual object recognition, the contrast enhanced by this luminance contrast illusion is thought to help in visually separating objects from their backgrounds [7]. Numerous studies have been conducted on the mechanism of the luminance contrast illusion in humans, and it has often been suggested that it is due to lateral inhibition in the photoreceptor cells of our retinas [8,9]. Lateral inhibition is the process by which photoreceptor cells, excited by a stimulus, suppress the activity of neighboring cells, thereby enhancing the changing part of the stimulus. On the other hand, it has also been reported that there were other cases where contrast was enhanced, even in two non-adjacent areas, which could not be explained by lateral inhibition alone, suggesting the existence of another mechanism in which luminance contrast is enhanced at the higher level of luminance judgment processing in the brain [6,10]. When humans identify the difference in luminance between the target and the background, the brain may emphasize this difference, resulting in the enhancement of the contrast. The luminance contrast illusion has been reported to occur in non-human animals such as chimpanzees [11], macaque monkeys [12], birds [13], fish [14,15], and insects [16,17,18], suggesting that the enhancement of contrast via this illusion may be important for visual object recognition in a wide range of species.

Cetaceans (dolphins, porpoises, and whales) are highly adapted to the underwater environment, which is very different from the terrestrial environment. In the underwater environment, sound information is greatly transmitted and can travel over hundreds and sometimes thousands of kilometers [19]. Cetaceans have acquired excellent auditory abilities, which are thought to be associated with these superiorities of sound information in water [20]. In particular, most toothed whales (odontocetes) have an ability called ‘echolocation’, enabling them identify the distance, size, shape, and even material of objects at a distance in front of the subject animal by using sounds as a biological sonar [21,22]. These auditory abilities have been studied extensively as an example of how differences in living environments have influenced the evolution of cognitive abilities. On the other hand, there has not been enough research focused on their visual abilities. Cetaceans’ visual acuity in water has been reported to be 8–14 arcmin [23], which is the same as, or below, that of many terrestrial mammals [24]. In addition, cetaceans lack the common dichromatic color vision achieved by two types of cones typical of many terrestrial mammals [23], making it difficult for cetaceans to distinguish colors. These visual abilities in cetaceans are likely related to the low dominance of visual information in the underwater environment; light is strongly affected by scattering, absorption, and attenuation by water [25]. The amount of light decreases with the water depth, and transparency depends greatly on water turbidity. In addition, absorption in water varies with the wavelength of the light; red light is absorbed much more readily than blue light. Nevertheless, observations in the wild [26,27,28] and behavioral experiments in captivity [29,30,31,32,33,34,35,36,37] have shown that cetaceans make effective use of information obtained through vision in their daily lives. Therefore, for cetaceans with neither high visual acuity nor color vision, contrast may be one of the few visual cues available.

However, very few studies have investigated how cetaceans perceive contrast. Murayama [38] conducted an experiment in which a captive bottlenose dolphin was asked to discriminate contrast under various ambient light conditions. The subject dolphin was required to choose a white plastic board with a black circle from a counter-presented board with no circle. The results showed that, even when there was sufficient ambient light, the discrimination performance varied depending on the magnitude of the contrast. On the other hand, this dolphin was able to discriminate objects even under relatively low illumination (0.8 lux). Thus, it was suggested that dolphins have the visual ability to perceive contrast regardless of the surrounding illumination. The strong photopic ability of cetaceans in low illumination is contributed by the high percentage of rods occupying 99% of the retina [39]. Cetaceans living in the underwater environment frequently experience drastic changes in the surrounding illumination due to their own movements. Their surroundings are bright when they are swimming along the surface of the water and become darker as the water depth increases. Therefore, it is possible that contrast—the difference in luminance between an object and its background—may be a more reliable visual cue for cetaceans than the luminance of the object itself. It is suggested that contrast, which is available regardless of the ambient light, plays an important role in visual object recognition in cetaceans. Furthermore, in the underwater environment, their visual worlds are assumed to have a more uniform background than in the terrestrial environment because the lower light transmission results in a shorter visible distance. It is possible that the enhancement of contrast via the luminance contrast illusion plays an important role in detecting objects visually from such backgrounds.

The research aim of this study was to examine experimentally whether the enhancement of contrast via the luminance contrast illusion could be observed in killer whales. Luminance discrimination tasks were conducted on two captive killer whales that were required to compare the luminance of two targets presented in monitors through an underwater window and choose the brighter one with their rostrums against the acrylic glass. In most of the general studies examining the luminance contrast illusion in humans and non-human animals, experiments were conducted in a situation where two target areas were surrounded by inducer areas of different luminance; one target was surrounded by a darker inducer, and the other was surrounded by a lighter inducer. However, we could run a limited number of trials per day due to the circumstances regarding housing and exhibition in the aquarium. Thus, we conducted experiments under the condition wherein the two targets were always surrounded by the inducer areas of the same luminance, so that the enhancement of the contrast via the illusion could be confirmed while reducing the overall number of trials. After the baseline training, in which both targets were surrounded by black or white inducer areas, a new condition of gray inducer areas was added as the test condition. If the subjects experienced the enhancement of contrast via the luminance contrast illusion as humans do, then, for the gray inducer condition, the correct target would appear brighter than it was and the incorrect stimulus would appear darker. Thus, it was expected that the apparent difference in luminance between the two targets would be larger than the actual difference. On the other hand, in the black inducer condition, both targets looked brighter, and it was expected that the apparent difference in luminance would not be changed. The same was true for the white inducer condition. Therefore, the following prediction was made: ‘the correct response rates for the gray inducer condition will exceed those for the black and white inducer conditions’. Furthermore, from the previous human studies [40,41,42], it is known that the size ratio of target areas to inducer areas affects the magnitude of the illusion, and that the smaller the target areas are relative to the inducer areas, the stronger the illusion becomes. Thus, to investigate the effect of the ratio of the size of the target areas and inducer areas on the occurrence of the illusion, Experiment 1 was conducted with 25 cm × 25 cm target areas and Experiment 2 was conducted with 10 cm × 10 cm target areas.

## 2. Materials and Methods

### 2.1. Subjects

Two killer whales (*Orcinus orca*), housed at the Port of Nagoya Public Aquarium in Aichi Prefecture, Japan, were used as the subjects of this study. One individual was an adult female named Lynn (8 years old), and the other was a sub-adult male named Earth (12 years old). Both individuals were born in captivity and were related. Although the operant conditioning using hand signs and whistle sounds was routinely conducted in this aquarium, these individuals had never previously undergone any cognitive experiments. All training and experiments were conducted when these two individuals were in different pools, so that they could not see each other. They were not food-deprived during the period of experimentation. The experimental procedure for the killer whales was approved by the Port of Nagoya Public Aquarium Committee and the Animal Welfare and Care Committee of the Center for the Evolutionary Origins of Human Behavior, Kyoto University (No. 2022-076).

### 2.2. Apparatus

**Figure 1:**
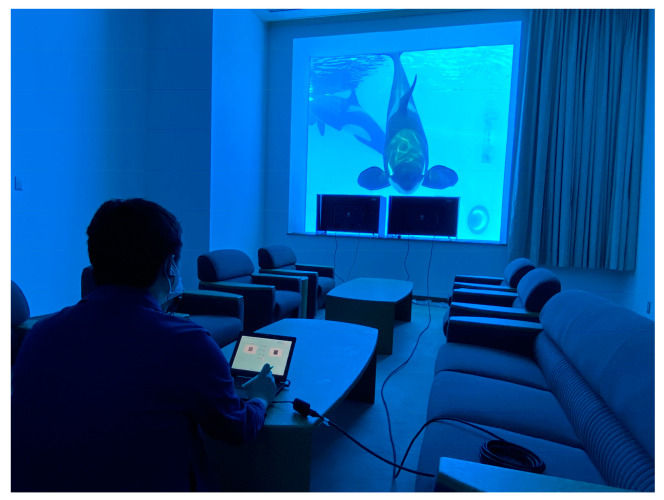
The experimental setup. Two monitors were placed 10 cm apart in front of the underwater window, and the experimenter (E2) manipulated the visual stimuli using a laptop 5 m behind the window. The subjects were required to compare the two targets presented on the monitors through an underwater window and touch the brighter one with their rostrum against the acrylic glass.

All training and experiments were conducted in an outdoor pool (elliptical shape, with dimensions of 34 × 11 m and 9 m in depth) with a west-facing underwater acrylic window (square, 3 × 3 m) located at a depth of 5 m. Two monitors (94 cm width × 53 cm height, I-O DATA, EX-LD4K432DB) were placed 40 cm away from the acrylic glass and 10 cm apart so that the experimenters could see which monitor was chosen through the gap between the monitors (Figure 1). As all experimental sessions were conducted during the daytime, each monitor was surrounded by a black sunshade to prevent sunlight from shining directly onto the screen. The laptop controlling the two monitors was placed 5 m behind the window in order to reduce the likelihood of the experimenter’s movements being used as a hint for the subject’s choice.

### 2.3. Procedure

The experiment was conducted in a situation wherein the subjects moved back and forth between the poolside and a position in front of the underwater window on the opposite side. These killer whales had originally learned one behavior during husbandry training: when they received a hand sign from the trainer at the poolside, they moved to the front of the underwater window and touched the acrylic glass against a target consisting of a white ball on the end of a black stick with their rostrum and returned to the trainer at the poolside after hearing a whistle sound. In this study, the training was based on the application of this behavior.

All experimental events were regulated by three experimenters. One experimenter (E1) at the poolside gave a hand sign as the start signal for the subject, and they then directed it to the underwater window. At the same time, the second experimenter (E2) in the room adjacent to the underwater window started the presentation of the visual stimuli, and the third experimenter (E3) held the whistle in his mouth and waited for the subject to approach the window. The subject’s body stopped approaching the window when it touched the acrylic glass with the rostrum. Since the subjects could not directly touch the visual stimuli presented through the glass, E3 visually determined whether the indirect touch with the rostrum was performed within the frame of either the left or right monitor, which was considered as a choice behavior. It was assumed that it would have been difficult for the subjects in the bright outdoor pool to visually observe these two experimenters, E2 and E3, who were located 5 m behind the monitors in the dark room. Furthermore, since they did not take any action after the subject had left the poolside and until the choice behavior, it is unlikely that the subjects obtained hints for their choices from the movements of these two experimenters. When the subject chose the correct answer, E3 blew a whistle and E2 finished the presentation of the stimuli. Then, E3 communicated, through a wireless device, the subject’s responses to E1, who was waiting at the poolside. Then, E1 gave one or two pieces of fish to the subject as a reward, so that the size of the reward given was equal per trial. When the subject chose the wrong answer, the presentation of the stimuli was finished with neither a whistle nor a reward. This series of events, from the start signal to the feedback, was regarded as one trial. The interval between the trials was defined as the time from which the subject returned to the poolside and rested in front of E1 until the next start signal was given, which was set to approximately 5 s.

If the subject left the poolside for any reason, then E1 waited for the subject to return and only then gave the start signal to initiate a trial. If the experimenter judged that the subject’s motivation was reduced due to consecutive incorrect answers, then the subject underwent a training event, such as the ‘fin swing’, unrelated to the experiment. After being confirmed as having performed the task in a calm state, it was rewarded. Continuing the trials despite low motivation due to consecutive incorrect answers would have increased the risk of undesirable behaviors such as extreme side bias and escape from the experiments. Therefore, we arranged for the subjects to reset their mood by providing them with simple training and reward fish, so that they could tackle the next trial with high motivation. One session consisted of 12 trials, and the subjects received one or two sessions in a day, with an inter-session interval of at least 5 min.

### 2.4. Stimuli

**Figure 2:**
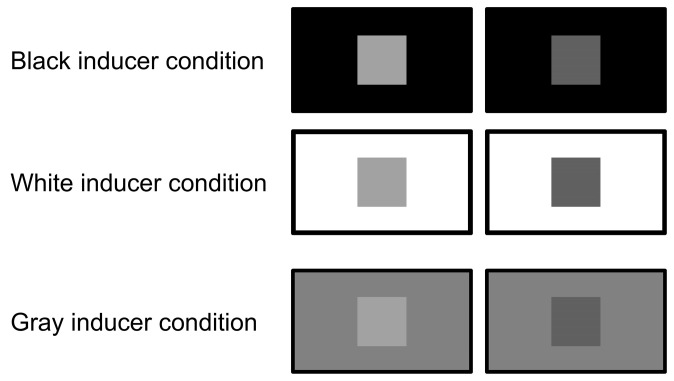
The visual stimuli used in Experiment 1. The target area was a square displayed in the center of each monitor, and the size was set to 25 cm width × 25 cm height. The subjects were trained to choose the brighter target. In this figure, the left targets are correct (luminance setting, 160) and the right targets are incorrect (luminance setting, 96), and the difference level of these conditions is ‘64’. The inducer area was a full screen surrounding the target area, and the size was 94 cm width × 53 cm height. In the baseline training sessions, two conditions were used: the black inducer condition (luminance setting, 0) and the white inducer condition (luminance setting, 255). The gray inducer condition (luminance setting, 128) was presented only in the test sessions.

**Figure 3:**
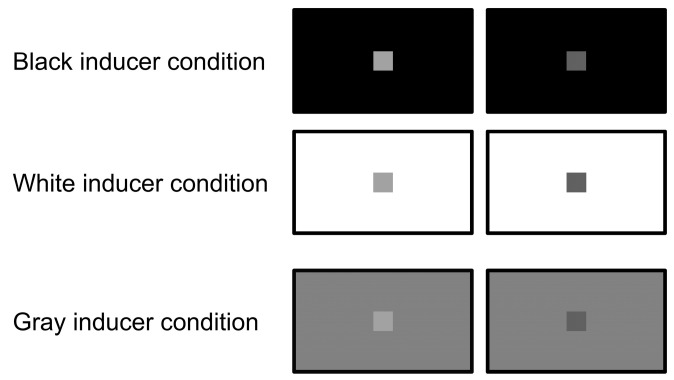
The visual stimuli used in Experiment 2. The size of the target areas was set to 10 cm width × 10 cm height. In this figure, the left targets are correct (luminance setting, 160) and the right targets are incorrect (luminance setting, 96), and the difference levels in these conditions are ‘64’. In the baseline training sessions, two conditions were used: the black inducer condition (luminance setting, 0) and the white inducer condition (luminance setting, 255). The gray inducer condition (luminance setting, 128) was presented only in the test sessions.

All visual stimuli were controlled by a custom program written in Microsoft Visual Studio 2019 (Microsoft Cooperation, Redmond, WA, USA). The refresh rate of the monitors was 60 Hz. The stimulus presented on each monitor was divided into a target area displayed in the center of each monitor and an inducer area surrounding the target area (Figure 2 and Figure 3). The luminance control was performed using the RGB (red, green, and blue) color model. This model defines colors as the combination of three values in 256 steps from 0 to 255. When these three values are the same, achromatic colors are output; ‘black’ was set to a value of (0, 0, 0) and ‘white’ was set to a value of (255, 255, 255). All 256 steps of achromatic color output by the RGB color model were used as the luminance intensity of the visual stimuli, and the value was called the ‘luminance setting’ in this study. The actual luminance value at each luminance setting was measured at the surface of the monitor using a colorimeter (Konica Minolta, Tokyo, Japan, CS-100A), between 0.35 cd/m^2^ at the luminance setting of ‘0’ and 161 cd/m^2^ at the luminance setting of ‘255’ (see Figure S1 for more details). The luminance settings of the two targets were selected so that they were separated by the same value, centered at 128 (in the middle of 256 steps). For example, when the luminance setting of one target was 144 (16 higher than 128) and that of the other one was 112 (16 lower than 128), the difference in luminance between the two targets was ‘32’; this value was called the ‘difference level’ and was used as the index for the difficulty level of the trial. The smaller this value, the smaller the difference in luminance between the two targets and thus the more difficult the task.

### 2.5. Experiment 1 (25 cm × 25 cm)

In Experiment 1, the size of the target areas was set to 25 cm × 25 cm. In the baseline training sessions, the subjects were trained to choose the brighter target (the one with the higher luminance setting) under two conditions: the black inducer condition (where the luminance setting of the inducer area was 0) and the white inducer condition (where the luminance setting of the inducer area was 255). Initially, the baseline training started at the difference level of ‘128’ (192 vs. 64). When the subjects maintained a steady 80% correct rate, we regarded them as having completed the training for the current difference level and moved on to the next difference level. This criterion was decided on by the experimenters, and it was based on the fact that it was statistically significant for the subject to have achieved 20 correct responses out of the previous 24 trials (binomial test, *p* = 0.0015) and on the experimenter’s judgement that an 80% correct rate was high enough to maintain the subject’s motivation. When the subjects reached the baseline of 80% at the difference level ‘64’ (160 vs. 96) for both the black and white inducer conditions, they moved on to the test sessions. The individual named Lynn took 1909 trials over 127 days and the other individual named Earth took 1396 trials over 83 days to move on to the test sessions.

In the test sessions, the new condition of the gray inducer, in which the luminance setting of the inducer area was 128 (centered value between 0 and 255), was introduced, and the correct response rates were examined for the black, white, and gray inducer conditions. In Experiment 1, a total of 240 trials (20 trials × 3 inducers × 4 difference levels) were conducted for each subject. Based on their proficiency, the four most appropriate difference levels were selected for each subject (‘96’, ‘64’, ‘48’, and ‘32’ for Lynn and ‘64’, ‘48’, ‘32’, and ‘24’ for Earth). All trials including three inducer colors and four difference levels were presented in a random order, and the correct target was also randomly presented on either the left or right monitor.

### 2.6. Experiment 2 (10 cm × 10 cm)

In Experiment 2, in order to verify the effect of the size ratio of the target and inducer areas on the occurrence of the illusion, the size of the target areas was set to 10 cm × 10 cm, smaller than the 25 cm × 25 cm in Experiment 1. Most of the experimental methods were the same as in Experiment 1. In the baseline training sessions, the subjects were trained to choose the brighter target under two conditions: the black and the white inducers. Initially, the baseline training started with the difference level of ‘128’ (192 vs. 64). When the subjects maintained a steady 80% correct rate, we regarded this as an indication that they had finished training for that difference level, and we moved on to the next level. When the subjects had reached the baseline of 80% at the difference level ‘64’ (160 vs. 96) in both the black and white conditions, they moved on to the test sessions. Lynn took 1040 trials over 40 days and Earth took 630 trials over 27 days to move on to the test sessions.

In the test sessions, the gray inducer condition was introduced, and the correct response rates were examined for the black, white, and gray inducer conditions. Since it was determined from Experiment 1 that 20 trials for each condition was too low a number and they might have been affected by data fluctuations, 40 trials were conducted for each condition in Experiment 2. In total, 480 test trials, 40 trials × 3 inducers × 4 difference levels, were conducted for each subject in Experiment 2. Based on their proficiency, the four difference levels, ‘64’, ‘48’, ‘32’, and ‘24’, were selected for both Lynn and Earth.

### 2.7. Statistical Analysis

Two types of statistical analysis were performed on the obtained correct response rates. The first was the binomial test, which was used to evaluate whether each condition was significantly correct or not. In Experiment 1, when the correct response rate was more than 75% (15 correct results out of 20 trials), it could be considered significantly correct (binomial test, *p* = 0.041). In Experiment 2, when the correct response rate was more than 67.5% (27 correct results out of 40 trials), it could be considered significantly correct (binomial test, *p* = 0.038). Secondly, based on the prediction that the correct response rates in the gray inducer condition would exceed those for the black and white inducer conditions, one-sided chi-squared tests were employed to examine whether differences in the inducer affected the correct response rates at each difference level. Here, the Bonferroni correction was applied to adjust the *p*-values for multiple comparisons across the three conditions.

## 3. Results

### 3.1. Experiment 1 (25 cm × 25 cm)

#### 3.1.1. Lynn

**Figure 4:**
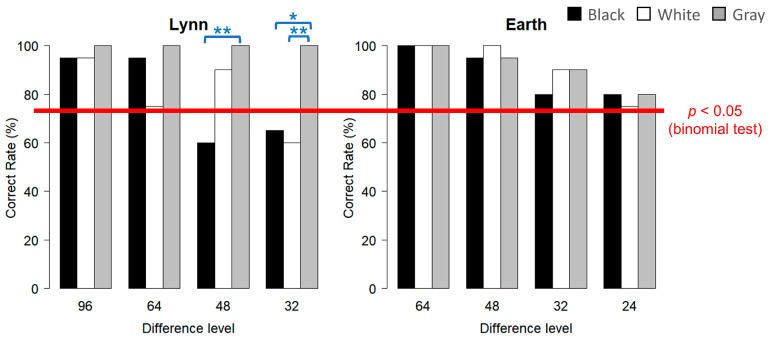
The results in terms of correct response rates in Experiment 1 (the **left** graph is for Lynn; the **right** graph is for Earth). The colors of the bars indicate the three inducers: black, white, and gray. The ‘difference level’ on the horizontal axis refers to the difference in the luminance setting between the two targets; the smaller this value, the greater the difficulty. The red line indicates the results of the binomial test; if the correct response rate is more than 75% (15 correct results out of 20 trials), it can be considered significantly correct (*p* < 0.05). The blue lines and asterisks placed between two bars indicate the results of the chi-squared test (* *p* < 0.05, ** *p* < 0.01).

The results regarding the correct response rates for each condition are plotted in Figure 4 (left graph). From the binomial test, the correct response rates for the gray inducer condition were significantly higher at all difference levels, while the correct response rates at the levels ‘48’ and ‘32’ for black and ‘32’ for white dropped to the chance level. From the multiple comparisons within each difference level, while no significant difference was found at the levels of ‘96’ or ‘64’, significant differences were found at the level of ‘48’ between black and gray (X-squared = 7.6562, df = 1, and *p* = 0.0085) and at the level of ‘32’ between black and gray (X-squared = 6.2338, df = 1, and *p* = 0.019) and between white and gray (X-squared = 7.6562, df = 1, and *p* = 0.0085).

#### 3.1.2. Earth

The results regarding the correct response rates for each condition are plotted in Figure 4 (right graph). From the binomial test, this subject could respond correctly at a significant level for the three inducer conditions at all four difference levels from ‘64’ to ‘24’. Similarly, the multiple comparisons within each difference level showed no significant differences among the black, white, and gray inducer conditions at any difference levels.

### 3.2. Experiment 2 (10 cm × 10 cm)

#### 3.2.1. Lynn

**Figure 5:**
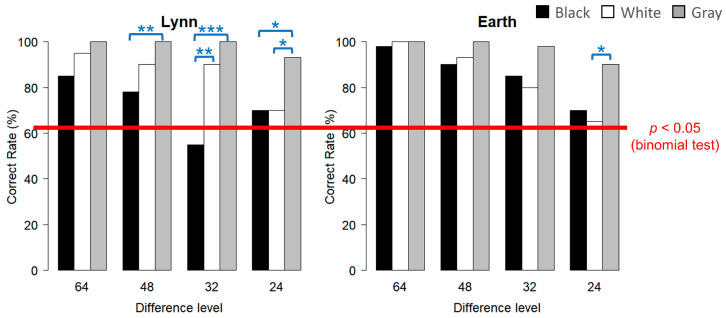
The results in terms of correct response rates in Experiment 2 (the **left** graph is for Lynn; the **right** graph is for Earth). The colors of the bars indicate the three inducers: black, white, and gray. The ‘difference level’ on the horizontal axis refers to the difference in the luminance setting between the two targets; the smaller this value, the greater the difficulty. The red line indicates the results of the binomial test; if the correct rate was more than 67.5% (27 correct results out of 40 trials), it could be considered significantly correct (*p* < 0.05). The blue lines and asterisks placed between two bars indicate the results of the chi-squared test (* *p* < 0.05, ** *p* < 0.01, *** *p* < 0.001).

The results regarding the correct response rates for each condition are plotted in Figure 5 (left graph). From the binomial test, the correct response rates for the gray inducer condition were significantly higher at all difference levels, while the correct response rate at the level of ‘32’ for the black inducer condition dropped to the chance level. From the multiple comparisons within each difference level, while no significant difference was found between white and gray at ‘64’, ‘48’, or ‘32’, significant differences were found between black and gray at the level of ‘48’ (X-squared = 8.0125, df = 1, and *p* = 0.0070) and at the level of ‘32’ (X-squared = 20.717, df = 1, and *p* < 0.001). At the difference level of ‘24’, there were significant differences both between black and gray (X-squared = 5.2513, df = 1, and *p* = 0.032) and between white and gray (X-squared = 5.2513, df = 1, and *p* = 0.032). Between black and white, a significant difference was only found for the level of ‘32’ (X-squared = 10.596, df = 1, and *p* = 0.0017).

#### 3.2.2. Earth

The results regarding the correct response rates for each condition are plotted in Figure 5 (right graph). According to the binomial test, this subject could correctly respond at a significant level for the three inducer conditions at all four difference levels, from ‘64’ to ‘24’. When performing multiple comparisons within each difference level, no significant difference in accuracy was found at the levels of ‘64’, ‘48’, and ‘32’. At the difference level of ‘24’, a significant difference was found between white and gray (X-squared = 5.8065, df = 1, and *p* = 0.024), while no significant difference was found between black and gray (X-squared = 3.8281, df = 1, and *p* = 0.076).

## 4. Discussion

In the current study, we aimed to examine experimentally whether the enhancement of contrast via the luminance contrast illusion could be observed in killer whales. Luminance discrimination tasks were conducted on two captive killer whales by presenting visual stimuli in monitors through an underwater window. The baseline training was conducted with the black and white inducer conditions, and then the gray inducer condition was added as the test condition. In Experiment 1, the size of the target area was set to 25 cm × 25 cm. From the binomial test, Lynn could correctly respond at a significant level in the gray inducer condition, while some correct response rates in the black and white inducer conditions dropped to the chance level. Furthermore, from the chi-squared test, Lynn showed significantly higher correct response rates in the gray inducer condition than black and white. These results for Lynn were consistent with the prediction of enhanced contrast through the luminance contrast illusion. On the other hand, Earth could correctly respond at a significant level in all inducer conditions, and no significant difference among these inducer conditions was observed. The reason for this individual difference may be associated with differences in their proficiency in luminance discrimination. While Lynn was correct in about 60% of cases at ‘32’ for black and white, Earth was correct in about 80% in these conditions. Although there was no clear reason for the difference in their proficiency and they experienced similar training, it is possible that the size of the target areas in Experiment 1 was large enough for Earth to discriminate the luminance of target areas without being affected by the inducer areas. In addition, the small number of 20 trials, conducted for each condition in Experiment 1, might have caused data fluctuations and affected the results. Therefore, in Experiment 2, the size of the target area was changed to 10 cm × 10 cm, and the trial number was increased to 40 trials for each condition. From the binomial test, Lynn could correctly respond at a significant level in almost all conditions, with the exception of only one condition. Nevertheless, from the chi-squared test, Lynn showed significantly higher correct response rates in the gray inducer condition than in black and white, which was consistent with Experiment 1. This suggested that the higher correct response rates in the gray inducer conditions in Experiments 1 and 2 were not attributed to data fluctuations but to the enhancement of contrast via the luminance contrast illusion. Similarly, Earth showed higher correct response rates in the gray than in the black and white inducer conditions. It should be noted, however, that only one combination showed a significant difference in the chi-squared test, suggesting that even the increased number of 40 trials was insufficient to detect a significant difference. Through these two experiments, it is suggested that the enhancement of contrast via the luminance contrast illusion could be observed also in killer whales, although there were some individual differences.

However, there is the possibility that these results were not due to the enhancement of contrast via the luminance contrast illusion but due to the use of the inducer areas as a criterion for luminance discrimination. In most of the general studies examining the luminance contrast illusion in humans and non-humans, the experiments were conducted in a situation wherein two target areas were surrounded by inducer areas of different luminance. In this study, on the other hand, the two targets were always surrounded by black, white, or gray inducer areas of the same luminance. The luminance settings of the two targets were set so that the average was always 128, so the smaller the value of the difference level, the more closely the luminance settings of the two targets approached 128, i.e., that of the gray inducer. Therefore, in the gray inducer condition, one target was brighter than the inducer and the other was darker than the inducer, making discrimination easier. On the other hand, the luminance settings of 0 (black inducer) and 255 (white inducer) were so far from those of the two targets that the discrimination performance was not affected. In summary, the subjects might have been able to easily make choices in the gray inducer condition by using the luminance of the inducer areas as a criterion for luminance discrimination, without the enhancement of contrast via the luminance contrast illusion. Although it is difficult to rule out this possibility completely, some results indicate that the enhancement of contrast affected their performance. Firstly, in the gray inducer condition, it is suggested that the enhancement of contrast contributed to visually separating the target areas from the inducer areas. For example, at the difference level of ‘48’ (152 vs. 104), it was essential for the subjects to visually separate the correct target (luminance setting: 152) from the gray inducer (luminance setting: 128) to make the correct choice. In Experiment 2, the subjects showed nearly 100% correct response rates at the difference level of ‘48’ in the gray inducer condition, indicating that they were able to almost perfectly discriminate this difference of 24 (152 vs. 128) between the correct target and the inducer. On the other hand, the correct response rates dropped to 70% at the difference level of ‘24’ (140 vs. 116) in the black and white inducer conditions. The 8-bit color patterns used to control the luminance in this study did not show a linear relationship with the absolute amount of light. Therefore, although these two discriminations did not show exactly the same degree of difficulty, it is suggested that separating a target from the background was easier than luminance discrimination between two targets. This is consistent with one aspect of the luminance contrast illusion, whereby the enhanced contrast helps the observer to visually separate objects from the background [7]. Secondly, the fact that the individual named Earth showed no significant difference among the three inducer conditions in Experiment 1 (25 cm × 25 cm), while there was a significant difference in Experiment 2 (10 cm × 10 cm), also indicates that the enhancement of contrast was observed in the killer whales. These results suggest that the magnitude of the illusion was affected by the size ratio of the target and inducer areas, and this is consistent with previous studies on humans [40,41,42]. If the luminance of the inducer had been used as a criterion for luminance discrimination, it would not explain why the results were affected by a change in the size ratio of the target areas to the inducer areas. The result in which the magnitude increased with a smaller ratio of target areas to inducer areas is characteristic of the luminance contrast illusion.

This is the first study to provide evidence that the enhancement of contrast via the luminance contrast illusion occurs in cetaceans. Similarly to other terrestrial mammals, cetaceans are known to use two types of photoreceptor cells, cones and rods, to sense visual information. However, there are two differences compared to terrestrial mammals. Firstly, the rod cells, which contribute to mesopic and scotopic vision, occupy 99% of the retina, while the cone cells, which contribute to photopic vision, occupy only 1% [39]. Secondly, they have only one type of cone [23,25], whereas most terrestrial mammals have two types. Multiple types of cones enable color discrimination based on the difference in the amount of response from each cone; most mammals with two types of cones have biochromatic color vision, while primates, including humans, with three types, have trichromatic vision. Therefore, cetaceans, with monochromatic color vision, are said not to be able to distinguish colors, and this has been confirmed in behavioral experiments [43,44]. Since all experiments in the current study were conducted in front of an underwater window at a depth of 5 m and the surrounding illumination was in the range of 100–1000 lux, it is thought that these killer whales used cone-based photopic vision to discriminate luminance [25]. Therefore, even though they used only one type of cone, which was smaller than the number of rods, the enhancement of contrast via the luminance contrast illusion occurred in these killer whales, similarly to terrestrial mammals. For cetaceans with low acuity and monochromatic color sensing, it is suggested that contrast may be one of the most important cues for visual object recognition and may help them to extract the contours of objects. Previous human studies have reported that a similar luminance contrast illusion occurs in rod-based vision as in cone-based vision [45,46]. Further experiments with controlled light environments are needed to determine whether killer whales experience a similar enhancement in contrast in mesopic and scotopic vision with rods.

Through this study, it is revealed that killer whales have the visual ability to perceive contrast as enhanced by the luminance contrast illusion. For killer whales with neither high visual acuity nor color vision, contrast may be an important cue available. The luminance contrast illusion has also been reported in fish such as goldfish (*Carassius auratus*) [14] and guppies (*Poecilia reticulata*) [15], which also live in water, suggesting that contrast may be an important cue for visual object recognition in the underwater environment. Their visual worlds in the underwater environment are assumed to have a more uniform background than in the terrestrial environment because the lower light transmission results in a shorter visible distance. Against this background, it would be very useful for animals living in water to extract the contours of an object using the contrast enhanced by the luminance contrast illusion and to notice its presence as soon as possible. This high sensitivity in noticing the presence of objects through vision can be a significant advantage for adaptation.

Several limitations can be found in the experiments in this study. The first limitation involves the small number of individuals and trials, as is true in most cognitive studies on cetaceans. The killer whales were kept in the aquarium, and time and labor limitations restricted the number of trials to 24 per day. In Experiment 1, 20 trials were conducted for each condition, and, in Experiment 2, 40 were conducted, which are very small numbers. Even so, given that it took almost a full year to train these two subjects for luminance discrimination tasks and complete the data collection for the tests, it was not realistic to increase the number of individuals and trials any further. The second limitation concerns the lack of strict control over the experimental conditions, especially the ambient illumination, even though this study included luminance discrimination tasks. This was mainly due to the fact that these experiments were conducted in an outdoor pool during the daytime, and the illumination in front of the underwater window fluctuated between 100 and 1000 lux. In order to exclude the effect of the ambient illumination as much as possible, black sunshades were used to surround the monitors, preventing direct sunlight from falling on them, and anti-reflective films were used to protect the monitor screens from reflections of light. Furthermore, the subjects were moved to the test sessions only after confirming that they could maintain a steady 80% correct response rate, even on sunny days with strong sunlight. However, the possibility that the ambient light conditions may have affected these results cannot be excluded completely. The third limitation is that it was impossible to measure the exact luminance of the stimuli from the subjects’ viewpoints in the water. They looked at visual stimuli displayed on monitors through 1 m of water between their eyes and the window, consisting of 10-cm-thick acrylic glass, and with 40 cm of air between the window and the monitors. Thus, the luminance of the stimuli that they saw was likely different from the luminance values measured while placed in the air. The fourth limitation is related to the refresh rates of the monitors and the critical flicker fusion frequencies of the subjects. In this study, visual stimuli were presented on monitors with a refresh rate of 60 Hz. Although the critical flicker fusion frequency in humans is known to be 50–90 Hz [47], there is no report of this in killer whales. For example, birds flying at high speeds are known to have higher critical flicker fusion frequencies than humans [48]. Therefore, killer whales, which can swim at high speeds in the water, may have a higher critical flicker fusion frequency than humans. If it were higher than the monitors’ refresh rate of 60 Hz, it is assumed that the monitors would have appeared to be blinking to them, and the possibility that this could have affected the results cannot be completely ruled out. The last limitation was the lack of strict control over the movements of the subjects. Because no restrictions were placed on their movements between the underwater window at a depth of 5 m and the poolside on the opposite side, they often made detours on their way and approached the window from an angle instead of from the front. Therefore, because we were unable to rigorously measure the distance from the stimuli when they made their choices and the time required in making their choices after they looked at the stimuli, we were unable to adopt these as indicators to discuss the results. Thus, while this study provides interesting findings regarding the visual perception of contrast in killer whales, it also raises several problems that need to be addressed in future studies examining the visual cognitive abilities of cetaceans.

## 5. Conclusions

We conducted luminance discrimination tasks with two captive killer whales to investigate whether the enhancement of contrast via the luminance contrast illusion could be observed in them. After baseline training, during which the targets were surrounded by black or white inducer areas, a test condition with gray inducer areas was added. In the two experiments, both individuals showed higher correct rates for gray than for black or white, although there were some individual differences. These results suggest that killer whales perceive contrast as enhanced via the illusion. For killer whales with neither high visual acuity nor color vision, it is suggested that contrast may be an important cue for visual object recognition and help them to extract the contours of objects from the background.

## Data Availability

Data are contained within the article.

## Supplementary Materials

The following supporting information can be downloaded at: https://www.mdpi.com/article/10.3390/ani15060793/s1, Figure S1: Luminance values of each luminance setting.
