# Supplementary figures and images for: Luminance Contrast Perception in Killer Whales (Orcinus orca)
Source: Animals (Basel). 2025 Mar 11;15(6):793. doi: 10.3390/ani15060793 (PMC11939218; doi:10.3390/ani15060793)

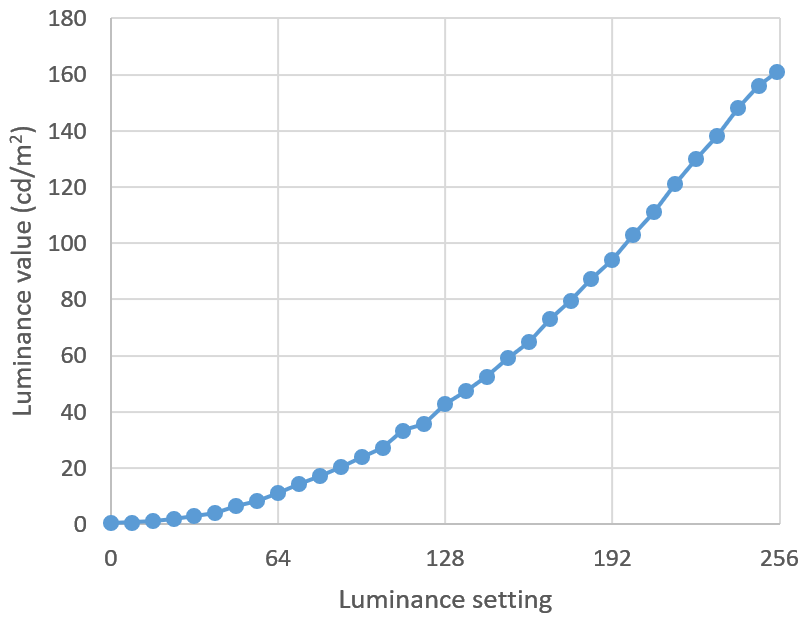

Supplement: Supplementary file 1 [file animals-15-00793-s001.zip › animals-3483627-supplementary.png]
